# Lemon Balm and Corn Silk Mixture Alleviates Metabolic Disorders Caused by a High-Fat Diet

**DOI:** 10.3390/antiox11040730

**Published:** 2022-04-07

**Authors:** Il-Je Cho, Joung-Hoon Shin, Beom-Rak Choi, Hye-Rim Park, Jeong-Eun Park, Seong-Hwa Hong, Young-Sam Kwon, Won-Seok Oh, Sae-Kwang Ku

**Affiliations:** 1 Department of Preparatory Korean Medicine, College of Korean Medicine, Daegu Haany University, Gyeongsan 38610, Korea; skek023@gmail.com or; 2 Department of Veterinary Surgery, College of Veterinary Medicine, Kyungpook National University, Daegu 41566, Korea; newyorkah@knu.ac.kr (J.-H.S.); kwon@knu.ac.kr (Y.-S.K.); 3 Nutracore Co., Ltd., Gwanggyo SK Viewlake A-3206, Beobjo-Ro 25, Suwon 16514, Korea; brchoi@nutracore.co.kr (B.-R.C.); hrpark@nutracore.co.kr (H.-R.P.); jpark@nutracore.co.kr (J.-E.P.); shhong@nutracore.co.kr (S.-H.H.); 4 Department of Veterinary Internal Medicine, College of Veterinary Medicine, Kyungpook National University, Daegu 41566, Korea

**Keywords:** corn silk (stigma of *Zea mays* L. fruit), high-fat diet (HFD), lemon balm (*Melissa officinalis* L.), 1:1 (*w*:*w*) mixture of lemon balm and corn silk extracts (M-LB/CS), metabolic disorders

## Abstract

We recently reported that varying combination ratios of lemon balm (*Mellissa officinalis* L.) and corn silk extracts (Stigma of *Zea mays* L. fruit) could reduce the obesity caused by a high-fat diet (HFD). The present study investigated the dose-dependent effect of a 1:1 (*w*:*w*) mixture of lemon balm and corn silk extracts (M-LB/CS) on HFD-mediated metabolic disorders and compared the effect with metformin. Oral administration of 50–200 mg/kg of M-LB/CS for 84 days significantly inhibited HFD-induced body weight gain, adipocyte hypertrophy, and lipogenic gene induction without affecting food consumption in mice. Biochemical analyses showed that M-LB/CS blocked abnormal lipid accumulation in the blood by escalating fecal lipid excretion. In addition, M-LB/CS prevented HFD-mediated pancreatic atrophy, decreased the number of insulin- and glucagon-immunoreactive cells, and inhibited increases in glycated hemoglobin, glucose, and insulin. Moreover, M-LB/CS also reduced hepatic injury, lipid accumulation, gluconeogenesis, and lipid peroxidation in parallel with the induction of AMP-activated protein kinase and antioxidant enzymes. Furthermore, M-LB/CS protected the kidney by inhibiting tubular vacuolation and reducing serum creatinine and blood urea nitrogen levels. The prophylactic effect of 100 mg/kg M-LB/CS-administration was comparable to that of metformin. Therefore, M-LB/CS may be an alternative option for managing obesity and its related metabolic disorders.

## 1. Introduction

Obesity is the dysregulated accumulation of body fat and is defined as a body mass index of 30 or higher. Obesity is often accompanied by high triglycerides, low high-density lipoprotein (HDL) complex, high blood pressure, and high glucose. When three or more symptoms are present at the same time, the individuals are diagnosed as having metabolic syndrome [1]. It has been estimated that about 20–25 percent of the adult population in the world has metabolic syndrome [2] and the prevalence of metabolic syndrome continues to escalate, especially in countries with excessive westernized diets and sedentary lifestyles. Although several approved medications can help to manage these complications, weight loss through exercise and a balanced diet to minimize unwanted side effects from the approved drugs has been suggested as a priority for overcoming metabolic syndrome.

Oxidative stress, an imbalance state between the reactive oxygen species (ROS) generating and scavenging systems, is considered a major etiology associated with obesity-mediated metabolic disorders [3], especially the high influx of dietary (saturated) fats that generates ROS during mitochondrial β-oxidation and facilitates lipotoxicity in the peripheral tissues (e.g., the liver, pancreas, and kidney). In addition, high fat intake exacerbates tissue injury by inducing low-grade systemic inflammation through microbial infiltration from a permeabilized intestinal barrier and the secretion of tumor necrosis factor-α from adipose tissue [4,5]. Moreover, macrophages that have engulfed oxidized low-density lipoprotein (LDL) complex produce foam cells, and the circulatory system is disturbed by the resulting accumulating plaque inside the arterial endothelium [6]. In this regard, medicinal herbs with excellent antioxidant activity can be consumed continuously without serious side effects, which has attracted great attention in the management of obesity-related metabolic disorders.

Of the valuable reservoirs of medicinal herbs for reducing oxidative stress, lemon balm (*Melissa officinalis* L., Lamiaceae family) is a fragrant herb and has been traditionally used for managing mental, cognitive, cardiovascular, and respiratory disorders in Europe and Central Asia [7]. In addition, corn silk (the style and stigma of *Zea mays* L. fruit) has been traditionally ingested for treating kidney, prostate, and urinary diseases in Asia and the Americas [8]. Diverse flavonoid derivatives (e.g., luteolin, naringin, hesperidin, maysin, and methoxy flavone), volatile compounds (e.g., geranial, neral, citronellal, geraniol, terpinol, citronellol, 6,11-oxidoacor-4-ene, pinocamphone, eugenol, neo-iso-3-thujanol, and sabinene hydrate), triterpenoids (e.g., usolic acid and oleanolic acid), and phenolic acids (e.g., rosmarinic acid and caffeic acid) have been isolated from both parental herbs [7,8]. Based on their potent antioxidant activity, lemon balm and corn silk have been reported to have the potential to prevent (or treat) a variety of diseases, including the metabolic complications of obesity [7,8,9,10,11,12,13].

In the process of developing a potent herbal mixture for managing obesity, we recently investigated the effect of mixtures consisting of lemon balm and corn silk extracts in varying combination ratios on high fat diet (HFD)–induced obesity in mice [14]. We found that 1:1, 1:2, 2:1, and 4:1 (*w*:*w*) mixture ratios of lemon balm and corn silk extracts (200 mg/kg each) significantly inhibited HFD-mediated body weight gain, hypertrophy of adipocytes, and abnormal blood lipid profiles. In addition, the anti-obesity effects of administering these combination ratios of the mixtures were greater than those by single herbal extracts alone. Moreover, the prophylactic effects of a 1:1 (*w*:*w*) mixture of lemon balm and corn silk extracts (M-LB/CS) were the most potent of the various combination ratios tested. To further extend our knowledge of M-LB/CS, the present study aims to explore the dose-dependent effects of M-LB/CS on HFD-mediated metabolic disorders. Furthermore, the prophylactic effect of M-LB/CS was compared to that of metformin (Met), an effective anti-obesity and anti-diabetic drug for type 2 diabetes [15].

## 2. Materials and Methods

### 2.1. Measurement of Flavonoid Content in M-LB/CS

Lemon balm extract and corn silk extract supplied by Evear Extraction (Coutures, France) and Xi’an Sost Biological Sciences and Technology Co., Ltd. (Xi’an, China) were directly mixed in a weight ratio of 1:1 and dissolved in water to prepare M-LB/CS [14]. Flavonoid content was quantified according to the method published by Suh and colleagues (2020), with slight modifications [16]. Briefly, 100 µL of M-LB/CS was incubated with 20 µL 10% aluminum nitrate (Sigma-Aldrich, St. Louis, MO, USA), 20 µL of 1 M potassium acetate (Duksan company, Ansan, Korea) and 860 µL of 80% ethanol for 40 min. Absorbance at the wavelength of 415 nm was measured using a microplate reader (EnSpire^TM^; PerkinElmer, Waltham, MA, USA). Baicalein (Sigma-Aldrich, St. Louis, MO, USA) was used as the standard for generating the calibration curve, and the flavonoid content of M-LB/CS was expressed as mg baicalein equivalents per gram of dry weight.

### 2.2. Quantification of Rosmarinic Acid and Allantoin in M-LB/CS

Rosmarinic acid and allantoin in M-LB/CS were quantified using high-performance liquid chromatography (HPLC), as described previously [14,17]. Briefly, M-LB/CS or rosmarinic acid (Sigma-Aldrich, St. Louis, MO, USA) were dissolved in 50% methanol and filtered using a 0.45 µm syringe filter (Advantec, Tokyo, Japan). M-LB/CS (10 µL) was loaded onto a Zorbax Eclipse C_18_ HPLC column (size, 4.6 × 250 mm; pore size, 5 µm) (Agilent Technologies, Palo Alto, CA, USA) with mobile phase comprising 25:75 acetonitrile:0.1% acetic acid. Eluants from M-LB/CS were detected using a photodiode array detector (Agilent Technologies, Palo Alto, CA, USA) at the wavelength of 330 nm. After obtaining the standard curve from the peak area of some diluted rosmarinic acid solutions, the concentration of rosmarinic acid in M-LB/CS was calculated by interpolating the peak area showing the same retention time as that of rosmarinic acid. In addition, M-LB/CS or allantoin (Sigma-Aldrich, St. Louis, MO, USA) dissolved in distilled water were loaded onto an Asahipak NH2P-50 4E HPLC column (size, 4.6 × 250 mm; pore size, 5 µm) (Shodex, Tokyo, Japan) under a gradient solution containing water and acetonitrile. After detecting eluants at 200 nm, the concentration of allantoin in M-LB/CS was calculated using the allantoin standard curve.

### 2.3. Animal Model and Treatment

Experimental protocol using laboratory animals was reviewed and approved by Institutional Animal Care and Use Committee of Daegu Haany University (approval no. DHU2019-087). High-fat diet (HFD), which provides 45% of total calories as fat, was purchased from Research Diet (New Brunswick, NJ, USA) and normal fat diet (NFD) was from Purinafeed (Seungnam, Korea). Female SPF/VAF CrljOri:CD1 (ICR) mice (N = 48; 6 weeks old) supplied by OrientBio (Seungnam, Korea) were acclimated for 10 days in standard rearing conditions, as described previously [14,18]. For adapting mice to the research diet, all mice were fed either HFD (N = 40) or NFD (N = 8) for 7 days and then assigned to six groups (N = 8 for each group): NFD, group of mice fed only NFD; HFD, group of mice fed only HFD; HFD + Met, group of mice fed HFD in conjunction with 250 mg/kg Met (Wako, Osaka, Japan)-administration; HFD + M-LB/CS (200 mg/kg), group of mice fed HFD with 200 mg/kg M-LB/CS-administration; HFD + M-LB/CS (100 mg/kg), group of mice fed HFD with 100 mg/kg M-LB/CS-administration; HFD + M-LB/CS (50 mg/kg), group of mice fed HFD with 50 mg/kg M-LB/CS-administration. During the experimental period of 84 days, mice were fed HFD or NFD, and three different doses of M-LB/CS or Met were orally administered once a day. In the cases of the NFD or HFD groups, the same amount of distilled water was administered to mice instead of the drug. Mice were euthanized 24 h after the last drug administration, and blood, fat pad, pancreas, liver, and kidney were collected for the subsequent experiments.

### 2.4. Measurement of Body Weight and Relative Organ Weight

Body weight was measured on days 0, 1, 7, 14, 21, 28, 35, 42, 49, 56, 63, 70, 77, 83, and 84 (i.e., day 0, the first day of administering drug) using a balance (Precisa Instrument, Zürich, Switzerland). To minimize the weight differences due to feeding, mice were fasted for approximately 12 h on days 0 and 84. Body weight gain was calculated by subtracting body weight on day 0 from the weight on day 84. Relative organ weight was calculated as a percentage of the weight of the specific organ relative to the body weight on day 84.

### 2.5. Measurement of Food Consumption

Food consumption was determined by weighing the remaining diet feed 24 h after feeding 150 g of diet feed and dividing by the number of mice in each rearing cage. Food consumption was measured once a week, and the mean daily food consumption was calculated as the average of the values obtained from the experimental period.

### 2.6. Measurement of Body Fat Mass Density

Body fat mass density was measured by scanning euthanized animals using an InAlyzer dual X-ray absorptiometry (DEXA) (Medikors, Seungman, Korea), as described previously [14,17].

### 2.7. Histopathology

Preparation of tissue sections, hematoxylin and eosin (H&E) staining, Oil Red O staining, and immunohistochemistry were conducted, as previously reported [17]. Briefly, the abdominal fat pad attached to the muscularis quadrantus lumborum, the splenic lobe of the pancreas, the left lateral lobe of the liver, and the left kidney were fixed, embedded in paraffin, sectioned, stained with H&E and observed under light microscope (Eclipse 80*i*; Nikon, Tokyo, Japan). Mean diameter of 10 adipocytes, thickness of fat pad, number of islets in the pancreatic parenchyma, mean area of zymogen granules in the exocrine pancreas, mean diameter of 10 hepatocytes, and number of vacuolated tubules among 100 renal tubules were quantified using an automated image analyzer (*i*Solution FL 9.1, IMT *i*-solution Inc., Burnaby, BC, Canada). In addition, the hepatic tissue was dehydrated in 30% sucrose, cryo-sectioned, stained with Oil Red O, and then the percentage of Oil Red O–stained regions in the hepatic parenchyma was quantified. Moreover, serial sectioned pancreatic tissue was incubated with insulin- or glucagon-directed antibodies (Abcam, Cambridge, UK) and immunoreactive cells were detected using biotinylated secondary antibody, avidin-biotin-peroxidase, and 3,3′-diaminobenzidine peroxidase substrate (Vector Laboratory, Burlingame, CA, USA). The cells showing over 20% immunoreactivity in the restricted view field of the pancreatic parenchyma were counted as the number of immunoreactive cells.

### 2.8. Quantitative Polymerase Chain Reaction (qPCR)

After total RNAs extracted from the periovarian fat pad or liver were reverse-transcribed to cDNA, qPCR was conducted using StepOnePlus^TM^ Real-Time PCR System (Applied Biosystems, Foster City, CA, USA). Oligonucleotide sequences for amplifying specific genes are listed in Table 1. Relative levels of specific genes were quantified based on C_T_ value [19], and glyceraldehyde 3-phosphate dehydrogenase was used as an endogenous control.

### 2.9. Measurement of Blood and Fecal Biomarkers

In addition to plasma glucose level, levels of serum cholesterol, triglycerides, LDL, HDL, alanine aminotransferase (ALT), aspartate aminotransferase (AST), creatinine, and blood urea nitrogen (BUN) were measured using a DRI-CHEM NX500*i* (Fuji medical system, Tokyo, Japan). In addition, glycosylated hemoglobin in whole blood and insulin in serum were detected using Easy A1c HbA1c measuring system (Infopia, Anyang, Korea) and mouse insulin enzyme-linked immunosorbent assay kit (Alpco Diagnostics, Windham, NH, USA), respectively. On day 83, feces were collected at 8 h after the last drug administration, and lipids were extracted by adding chloroform and methanol. Levels of cholesterol and triglycerides in fecal lipids were calorimetrically determined using total cholesterol assay kit (Cell Biolabs, San Diego, CA, USA) and triglyceride assay kit (Cayman, Ann Arbor, MI, USA), respectively.

### 2.10. Measurement of Glucose 6-Phosphatase (G6pase) and Phosphoenolpyruvate Carboxykinase (PEPCK) Activities

0.3 g of the liver was homogenized in 0.1 M triethanolamine, 0.2 M ethylenediaminetetraacetic acid, and 2 mM dithiothreitol and then clarified by centrifugation at 1000× *g* for 15 min. Supernatant was further centrifuged at 10,000× *g* for 15 min for preparing liver homogenate. Enzyme activities for G6pase and PEPCK were determined as described previously [17,20]. Briefly, liver homogenate was incubated with glucose 6-phosphate, mutarotase, oxidized nicotinamide adenine dinucleotide (NAD), and glucose dehydrogenase. To measure G6pase activity, NADH generated from the reaction mixture was detected at 340 nm. In addition, the decrease in optical intensity at 340 nm was measured to examine PEPCK activity after incubation of liver homogenates with NADH, malate dehydrogenase, and phosphoenolpyruvate. The enzyme activities were normalized by protein concentration.

### 2.11. Measurement of Malondialdehyde and Antioxidant Capacities

The liver homogenate prepared in 0.01 M Tris-HCl (pH 7.4) was incubated with thiobarbiturate for 1 h at 95 °C, and optical intensity at 525 nm was measured to detect malondialdehyde. In addition, clarified liver homogenate was incubated with 5,5′-dithiobis(2-nitrobenzoic acid) after adding trichloroacetic acid, followed by measuring optical intensity at 412 nm to detect glutathione. Catalase activity was determined at 240 nm after incubating the liver homogenate with hydrogen peroxide. Of note, 1 unit of catalase was defined as the amount of enzyme capable of degrading 1 nM hydrogen peroxide for 1 min. Moreover, superoxide dismutase activity was measured at 560 nm after incubating the liver homogenate with xanthine, xanthine oxidase, and nitroblue tetrazolium. Of note, 1 unit of superoxide dismutase was defined as the amount of enzyme that can reduce initial optical intensity by 50% for 1 min. Levels of malondialdehyde, glutathione, catalase, and superoxide dismutase were normalized by tissue weight.

### 2.12. Statistical Analysis

All numerical results are expressed as mean ± standard deviation of eight mice. Samples with homogeneity variance were analyzed by One-Way ANOVA and Tukey’s range test to find means that are significantly different among experimental groups. Meanwhile, samples that violated the assumption of homogeneity of variance were analyzed by Welch’s ANOVA and Dunnett’s T3 test. All analyses were conducted using SPSS Statistics 18 (SPSS Inc., Chicago, IL, USA), and *P* values less than 0.05 were considered significant.

## 3. Results

### 3.1. M-LB/CS Prevents HFD-Mediated Weight Gain

**Figure 1:**
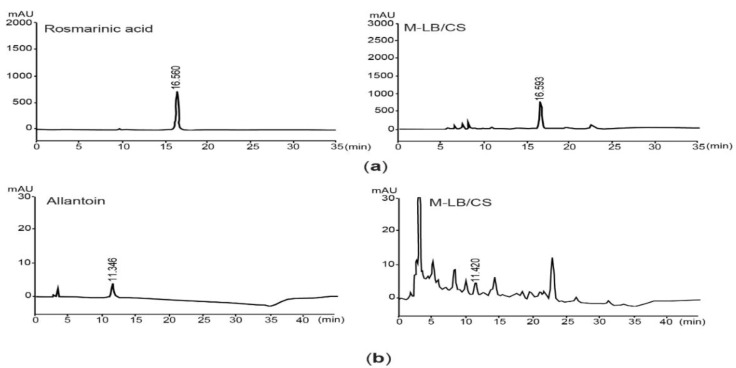
Quantification rosmarinic acid and allantoin in 1:1 (*w*:*w*) mixture of lemon balm and corn silk extracts (M-LB/CS). (**a**) HPLC chromatograms were detected at 330 nm after eluting rosmarinic acid (left) and M-LB/CS (right). (**b**) Chromatograms for allantoin (left) and M-LB/CS (right) were detected at 200 nm. The numbers in each chromatogram are retention times (min). HPLC, high-performance liquid chromatography.

Prior to exploring the beneficial effects of M-LB/CS against HFD-mediated metabolic disorders, we quantified the flavonoid content in M-LB/CS. We found that M-LB/CS had 89.01 ± 10.01 mg baicalein equivalents/g flavonoids. Next, since it was reported that rosmarinic acid and allantoin are bioactive compounds enriched by lemon balm and corn silk [14], those concentrations in M-LB/CS were assessed through HPLC analysis. By comparing the peak areas showing similar retention times to the standard compounds (Figure 1a,b), we found that the M-LB/CS used in this study contained 22.45 ± 0.11 mg/g of rosmarinic acid and 0.80 ± 0.04 mg/g of allantoin, respectively.

**Figure 2:**
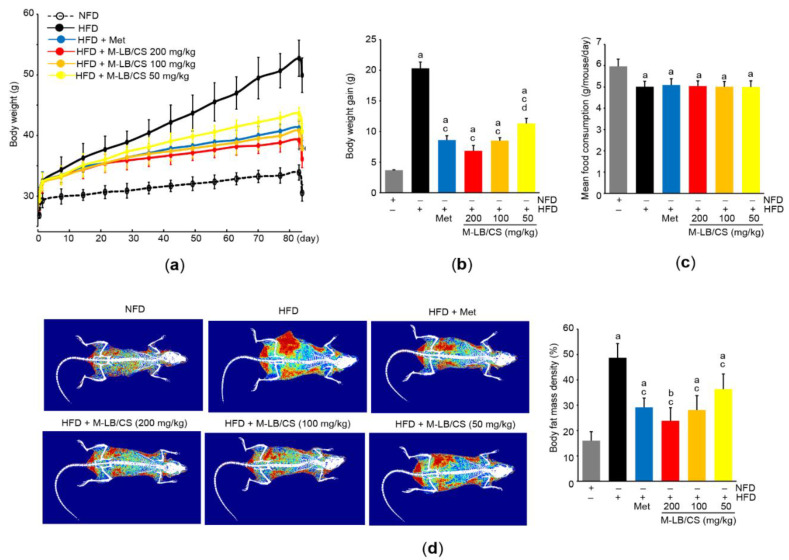
M-LB/CS prevents weight gain in mice fed high-fat diet (HFD). (**a**) Changes in body weight during the experimental period. Mice fed HFD were administered metformin (Met) or 50–200 mg/kg M-LB/CS for 84 days. Control mice were fed normal fat diet (NFD). (**b**,**c**) Body weight gain (**b**) and mean daily food consumption (**c**) were calculated as described in the Materials and Methods Section. (**d**) Representative images (left) and body fat mass density (right) were obtained from dual X-ray absorptiometry. The gray, black, blue, red, orange, and yellow bars in Figure 2b–d are NFD, HFD, HFD + Met, HFD + M-LB/CS 200 mg/kg, HFD + M-LB/CS 100 mg/kg, and HFD + M-LB/CS 50 mg/kg, respectively. ^a^ *p* < 0.01, ^b^ *p* < 0.05 versus NFD; ^c^ *p* < 0.01 versus HFD; ^d^ *p* < 0.05 versus HFD + Met.

During the 7-day adaptation period to the diet, the body weights of the mice fed the HFD were significantly increased compared with the mice fed the NFD (i.e., 26.71 ± 0.50 g in the NFD group versus 29.24 ± 1.64 g in the HFD group). Next, the mice were fed the HFD for 84 days with the administration of three different doses of M-LB/CS. NFD was supplemented for the control mice, and Met (250 mg/kg/day) was used as a reference drug. As compared with mice fed the HFD, significant reductions in body weight were seen from 28 days after the administration of Met, 200 mg/kg M-LB/CS, and 100 mg/kg M-LB/CS (i.e., day 0, the first day either M-LB/CS or Met was administrated). Significant decreases in body weight were noted from 35 days in the case of mice administered with 50 mg/kg M-LB/CS. During the experimental period, there were no statistical differences in body weights between Met and the 3 different doses of M-LB/CS (Figure 2a). Similarly, increases in body weight gain for 84 days were significantly decreased in mice administered 50–200 mg/kg M-LB/CS. There was no statistical difference in weight gain between mice administered 100–200 mg/kg M-LB/CS and Met (Figure 2b). To further explore whether M-LB/CS decreases body weight by reducing appetite, we monitored mean daily food consumption. Although the food consumption was significantly decreased in all mice supplemented with the HFD, there were no differences in food consumption among mice fed the HFD, HFD + Met, and HFD + 50–200 mg/kg M-LB/CS (Figure 2c). In parallel with body weight, results from DEXA scans obviously showed that the HFD-mediated increases in fat mass density of the entire body were significantly suppressed in mice administered 50–200 mg/kg M-LB/CS, and there were no differences in fat mass density between mice administered 50–200 mg/kg M-LB/CS and Met (Figure 2d).

### 3.2. M-LB/CS Reduces HFD-Mediated Hypertrophy of Adipose Tissue

**Figure 3:**
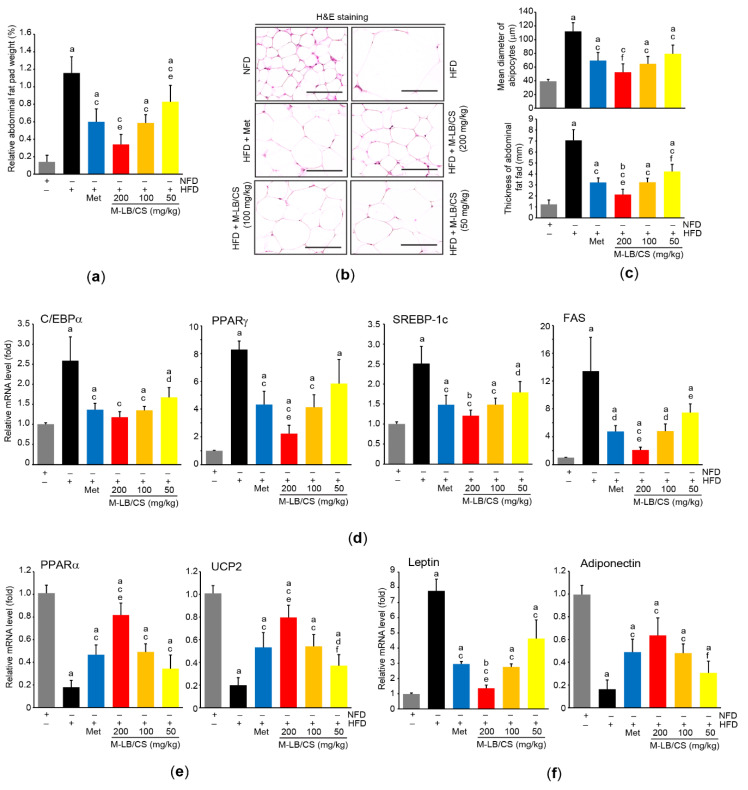
M-LB/CS reduces hypertrophy and fat storage in adipose tissue. (**a**) Relative weight of abdominal fat pad was determined as a percentage of abdominal fat weight relative to body weight. (**b**) Abdominal adipose tissue was stained with H&E. Scale bars indicate 80 µm. (**c**) Mean diameter of adipocytes (upper) and thickness of abdominal fat pad (lower) were measured using an automated image analyzer. (**d**–**f**) The mRNA levels of genes related to lipogenesis (**d**), lipolysis (**e**), and adipokine (**f**) in periovarian adipose tissue were analyzed by qPCR. The gray, black, blue, red, orange, and yellow bars in Figure 3a,c,d,e,f are NFD, HFD, HFD + Met, HFD + M-LB/CS 200 mg/kg, HFD + M-LB/CS 100 mg/kg, and HFD + M-LB/CS 50 mg/kg, respectively. ^a^ *p* < 0.01, ^b^ *p* < 0.05 versus NFD; ^c^ *p* < 0.01, ^d^ *p* < 0.05 versus HFD; ^e^ *p* < 0.01, ^f^ *p* < 0.05 versus HFD + Met. H&E, hematoxylin and eosin; qPCR, quantitative polymerase chain reaction.

We measured relative weight to explore the effect of M-LB/CS on the adipose tissue. Administration of 50–200 mg/kg M-LB/CS significantly inhibited the increase in relative weight of abdominal adipose tissue by HFD supplementation, and the magnitude of the reduction in relative weight of the tissue from administering 200 mg/kg M-LB/CS was greater than that from Met. However, there was no difference in the relative weight between mice fed 100 mg/kg M-LB/CS and those receiving Met (Figure 3a). Similarly, histopathological results from H&E-stained abdominal adipose tissue indicated that the three different doses of M-LB/CS significantly decreased the thickness of adipose tissue as well as the mean diameter of the adipocytes increased by the HFD. The reduction in adipocyte hypertrophy by 200 mg/kg M-LB/CS was more potent than that by Met (Figure 3b,c). Next, mRNA levels of specific genes associated with lipid metabolism were further quantified to explore molecular changes in the adipocytes (Figure 3d–f). As expected, the HFD significantly increased mRNA levels associated with lipogenesis (e.g., C/EBPα, PPARγ, SREBP-1c, and FAS) and inhibited the genes related to lipolysis and energy expenditure (e.g., PPARα and UCP2). However, the HFD-mediated abnormal changes in the mRNA levels of the aforementioned genes were significantly prevented by the administration of the three different doses of M-LB/CS, with the exception of PPARγ and FAS mRNAs in mice administered HFD + 50 mg/kg M-LB/CS. The magnitudes of the 200 mg/kg of M-LB/CS-mediated changes in PPARγ, FAS, PPARα, and UCP2 mRNAs were greater than those associated with Met (Figure 3d,e). In addition, the HFD inversely regulated mRNA levels of leptin and adiponectin, which is in parallel with previous reports [14,21,22]. Although 50 mg/kg M-LB/CS did not prevent the reduction of adiponectin mRNA in response to the HFD, the three different doses of M-LB/CS significantly minimized the aberrant expression of adiponectin and leptin mRNAs. Moreover, the reduction in leptin mRNA from administering 200 mg/kg of M-LB/CS was greater than that from Met (Figure 3f).

### 3.3. M-LB/CS Inhibits HFD-Mediated Abnormal Lipid Profiles

Next, we analyzed biomarkers to explore whether M-LB/CS can inhibit HFD-mediated abnormal lipid circulation. As expected, the HFD led to increased serum levels of total cholesterol, triglycerides, and LDL. On the contrary, the HFD significantly reduced serum HDL. However, the abnormal HFD-mediated changes in serum lipids were inhibited by administering the three different doses M-LB/CS. In particular, the magnitude of the reductions in serum cholesterol and triglycerides from administering 200 mg/kg M-LB/CS were greater than those from Met (Table 2). In addition, 100 and 200 mg/kg M-LB/CS significantly escalated the fecal excretion of cholesterol and triglycerides, while 50 mg/kg M-LB/CS could significantly increase fecal triglyceride excretion alone (Table 2).

### 3.4. M-LB/CS Protects against HFD-Mediated Pancreatic Injury

**Figure 4:**
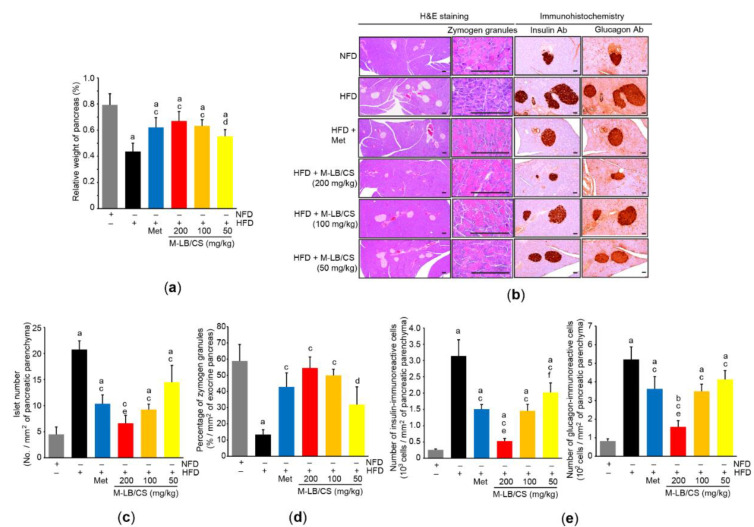
M-LB/CS alleviates hyperplasia of pancreatic islets. (**a**) Relative weight of pancreas was measured as described in Figure 3a. (**b**) Representative histological images. Tissue sections prepared from the pancreas were stained using either H&E (left) or antibodies to insulin and glucagon (right). Scale bars indicate 80 µm. (**c**–**e**) Islet number (**c**), zymogen granules (**d**), and insulin- and glucagon-immunoreactive cells (**e**) were counted using an automated image analyzer. The gray, black, blue, red, orange, and yellow bars in Figure 4a,c–e are NFD, HFD, HFD + Met, HFD + M-LB/CS 200 mg/kg, HFD + M-LB/CS 100 mg/kg, and HFD + M-LB/CS 50 mg/kg, respectively. ^a^ *p* < 0.01, ^b^ *p* < 0.05 versus NFD; ^c^ *p* < 0.01, ^d^ *p* < 0.05 versus HFD; ^e^ *p* < 0.01, ^f^ *p* < 0.05 versus HFD + Met. Ab, antibody.

Because the pancreas is an essential endocrine organ that regulates glucose homeostasis and a HFD is a well-known risk factor for diabetes mellitus [23], we further investigated the protective effects of M-LB/CS against HFD-mediated pancreatic injury. The HFD significantly decreased the relative weight of the pancreas, suggesting that the HFD induces atrophy of the pancreas. However, the three different doses of M-LB/CS significantly blocked the pancreatic atrophy. There were no statistical differences in relative weights of the pancreas between Met and M-LB/CS (Figure 4a). Histopathological examination of the pancreatic tissue indicated that HFD supplementation increased the number of pancreatic islets, while M-LB/CS-administration significantly prevented the increase in islet number in a dose-dependent manner. In particular, the reduction in islet number from 200 mg/kg M-LB/CS was greater than that seen in Met-administration (Figure 4b,c). On the contrary, the HFD significantly decreased the percentage of zymogen granules, specialized subcellular organelles in pancreatic acinar cells that regulate the secretion of digestive enzymes [24]. However, the three different doses of M-LB/CS significantly blocked the reduction of zymogen granules, and these effects were similar to that of Met (Figure 4b,d). Immunohistochemical staining of serially sectioned pancreatic tissue showed that the HFD significantly increased the number of insulin- and glucagon-immunoreactive cells in the pancreatic parenchyma, and these were significantly inhibited by administering the three different doses of M-LB/CS. The magnitude of the reduction in insulin- and glucagon-immunoreactive cells from 200 mg/kg M-LB/CS was greater than that from Met (Figure 4b,e). In parallel with the results of immunohistochemical staining, blood biomarkers also showed that M-LB/CS dose-dependently inhibited HFD-mediated increases in insulin, glucose, and glycosylated hemoglobin (Table 2).

### 3.5. M-LB/CS Prevents HFD-Mediated Hepatotoxicity by Ameliorating Lipid Accumulation and Oxidative Stress

Although there were no statistical differences in relative liver weight among the experimental groups (data not shown), the enzymatic activities of ALT and AST in the serum were significantly increased by HFD supplementation, which suggests that the HFD induces hepatotoxicity. However, the three different doses of M-LB/CS prevented the HFD-mediated increases in serum ALT and AST activities. In particular, the prophylactic effect on serum hepatotoxicity of 200 mg/kg M-LB/Cs was greater than that associated with Met (Table 2).

**Figure 5:**
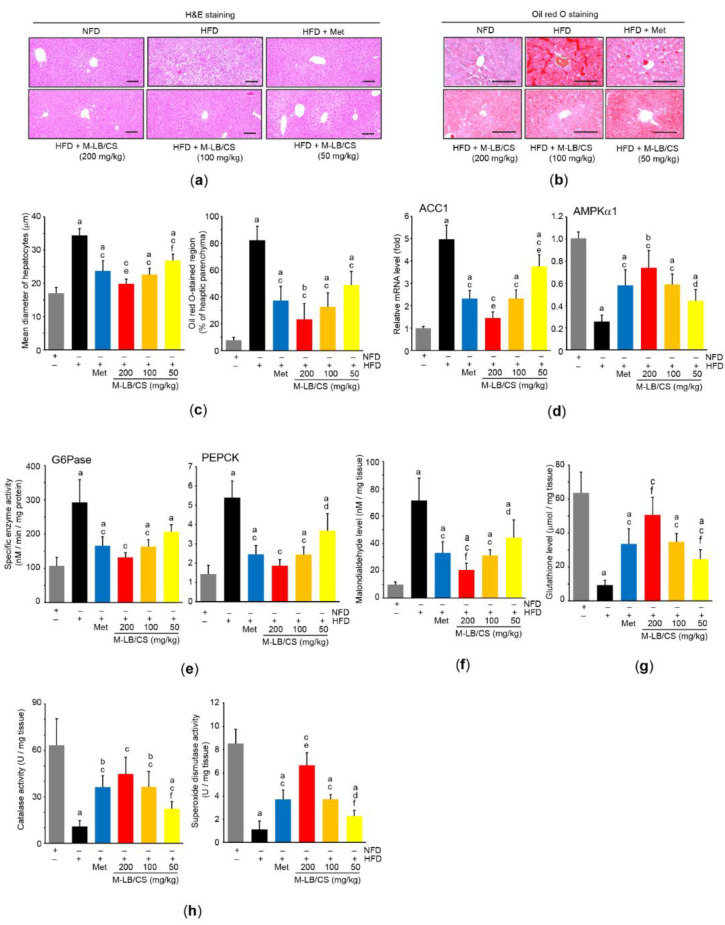
M-LB/CS mitigates abnormal hepatic changes induced by HFD. (**a**,**b**) Hepatic tissues were stained with either H&E (**a**) or Oil Red O (**b**). Scale bars indicate 80 µm. (**c**) Mean diameter of hepatocytes (left) and Oil Red O–stained regions in hepatic parenchyma (right) were counted using an automated image analyzer. (**d**) The levels of hepatic ACC1 (left) and AMPKα1 (right) mRNA were analyzed by qPCR. (**e**) Hepatic G6Pase and PEPCK activities were determined using a tissue homogenate and normalized by protein concentration. (**f**–**h**) The hepatic levels of malondialdehyde (**f**), glutathione (**g**), and catalase and superoxide dismutase activities (**h**) were assessed. The gray, black, blue, red, orange, and yellow bars in Figure 5c–h are NFD, HFD, HFD + Met, HFD + M-LB/CS 200 mg/kg, HFD + M-LB/CS 100 mg/kg, and HFD + M-LB/CS 50 mg/kg, respectively. ^a^ *p* < 0.01, ^b^ *p* < 0.05 versus NFD; ^c^ *p* < 0.01, ^d^ *p* < 0.05 versus HFD; ^e^ *p* < 0.01, ^f^ *p* < 0.05 versus HFD + Met. ACC, acetyl-CoA carboxylase; AMPK, AMP-activated protein kinase; G6Pase, glucose 6-phosphatase; PEPCK, phosphoenolpyruvate carboxykinase.

Histopathological observation of the hepatic tissue indicated that the HFD-mediated increase in hepatocyte diameter was dose-dependently decreased by administering M-LB/CS, and the magnitude of the reduction in the enlarged hepatocytes from 200 mg/kg M-LB/CS was more potent than that from Met (Figure 5a,c-left). In addition, the hepatic tissue stained with Oil Red O indicated that M-LB/CS significantly inhibited HFD-mediated hepatic lipid accumulation (Figure 5b,c-right). We further explored the effects of M-LB/CS on key regulators involved in hepatic lipogenesis. As expected, HFD supplementation significantly induced the mRNA level of ACC1, which converts acetyl-CoA to malonyl-CoA for engaging fatty acid synthesis [25]. However, the administration of the three different doses of M-LB/CS significantly reduced ACC1 induction, and the inhibitory effect of ACC1 mRNA of 200 mg/kg M-LB/CS was greater than that shown with Met. On the other hand, M-LB/CS-administration significantly prevented the HFD-mediated reductions in the mRNA levels of AMPKα1, an upstream negative regulator of ACC [25,26]. There were no statistical differences in the AMPKα1 mRNA levels of mice administered M-LB/CS and Met (Figure 5d).

In parallel with the results of blood glucose and glycosylated hemoglobin (Table 2), the HFD significantly increased the enzyme activities of G6Pase and PEPCK, which are major regulatory enzymes of gluconeogenesis [27]. However, M-LB/CS reduced both enzymatic activities, with the exception of G6Pase activity in the 50 mg/kg M-LB/CS treatment, which did not differ from that in mice supplemented with the HFD (Figure 5e).

To explore whether M-LB/CS can protect the liver by activating antioxidant systems, we primarily assessed the radical scavenging activity of M-LB/CS. Our supplementary result showed that M-LB/CS dose-dependently decreased the optical intensity raised by the radical 2,2-diphenyl-1-picrylhydrazyl (DPPH), and the IC_50_ of M-LB/CS for scavenging the DPPH radical was 65.93 ± 13.71 µg/mL (Supplementary Materials, Figure S1). In addition, M-LB/CS significantly reduced HFD-mediated increases in malondialdehyde (a marker of lipid peroxidation) in the hepatic tissue (Figure 5f). Moreover, the three different doses of M-LB/CS significantly blocked reductions in the hepatic antioxidant system, such as the reductions in the levels of glutathione, catalase, and superoxide dismutase in response to the HFD (Figure 5g,h). Finally, the magnitude of both reductions in lipid peroxidation and the activation of the antioxidant system from 200 mg/kg M-LB/CS were greater than those seen in response to Met, except for catalase activity (Figure 5f–h).

### 3.6. M-LB/CS Alleviates HFD-Mediated Kidney Injury

**Figure 6:**
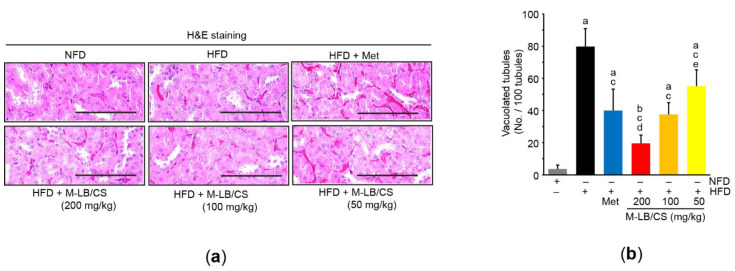
M-LB/CS blocks lipid accumulation in the kidney. (**a**) Representative images of kidney tissue after staining with H&E. Scale bars indicate 80 µm. (**b**) Vacuolated tubules were counted using an automated image analyzer. The gray, black, blue, red, orange, and yellow bars in Figure 6b are NFD, HFD, HFD + Met, HFD + M-LB/CS 200 mg/kg, HFD + M-LB/CS 100 mg/kg, and HFD + M-LB/CS 50 mg/kg, respectively. ^a^ *p* < 0.01, ^b^ *p* < 0.05 versus NFD; ^c^ *p* < 0.01 versus HFD; ^d^ *p* < 0.01, ^e^ *p* < 0.05 versus HFD + Met.

Although there were no differences in relative kidney weight among experimental groups (data not shown), the three different doses of M-LB/CS significantly reduced HFD-mediated increases in blood levels of creatinine and BUN. Reductions in blood biomarkers associated with kidney toxicity seen from 200 and 100 mg/kg M-LB/CS did not differ from those from Met (Table 2). In addition, histological observation of H&E-stained kidney tissue showed that M-LB/CS dose-dependently prevented the vacuolation of renal tubules induced by HFD supplementation, and the reduction of tubular vacuolation by 200 mg/kg M-LB/CS was more potent than that associated with Met (Figure 6a,b).

## 4. Discussion

We and others have reported that rosmarinic acid and allantoin are major compounds for assessing the quality of lemon balm and corn silk [14,17,28]. In addition, rosmarinic acid and allantoin are potent candidates to prevent obesity-mediated metabolic disorders by inhibiting lipogenesis and protecting organs [29,30,31,32,33,34]. Moreover, because flavonoids are representative bioactive compounds for promoting human health [35], we further quantified total flavonoid content and found that about 9% of M-LB/CS consisted of flavonoids (as baicalein equivalent). Throughout pharmacognostical analyses, a variety of flavonoids and their glycosides were identified in lemon balm and corn silk [7,8]. Of those, luteolin, apigenin, hesperetin, hesperidin, naringin, naringenin, isoquercitrin, rutin, and maysin have been suggested to inhibit HFD-mediated metabolic disorders [36,37,38,39,40,41,42,43]. Therefore, rosmarinic acid, allantoin, the aforementioned flavonoids, and other unidentified phytochemicals may collaboratively contribute to the prophylactic action of M-LB/CS against HFD-mediated metabolic disorders. Studies to elucidate bioactive phytochemicals can allow for a deeper understanding of the molecular mechanisms of M-LB/CS.

The present results show that administration of M-LB/CS dose-dependently inhibited weight gain and lipid accumulation in mice fed the HFD. More specifically, M-LB/CS regulated several essential genes associated with lipogenesis, lipid oxidation, and energy expenditure in adipose tissue. It has been reported that PPARγ in conjunction with C/EBPα triggers adipocyte maturation [44]. Together with SREBP-1c, PPARγ can stimulate the induction of lipogenic genes (e.g., FAS). In addition, it also promotes the uptake of fatty acids into adipose tissue to store triglycerides and facilitates the secretion of adipokines (e.g., leptin and adiponectin) [45,46]. On the contrary, PPARα induces the expression of several genes that contribute to fatty acid oxidation and thermogenesis (e.g., UCP) in the adipose tissue [45]. Interestingly, it was reported that lemon balm contains bioactive compounds that can bind to PPARγ, PPARα, and PPARβ/δ [47]. Furthermore, our previous report showed the modulating effect of PPARs by lemon balm extract was enhanced by its combination with corn silk extract [14]. Therefore, the present results suggest that PPARs regulation may be one of the mechanisms involved in the inhibition of adipocyte hypertrophy by M-LB/CS.

The present results showing the HFD increased the levels of glycated hemoglobin, glucose, and insulin in the blood and the abnormal expansion of pancreatic α/β cells consolidate the concept that the HFD induces hyperglycemia with insulin resistance [23,48]. In particular, because glycosylated hemoglobin is produced when erythrocytes are exposed to high concentrations of glucose, glycosylated the hemoglobin level represents the long-term history of blood glucose [49]. Thus, consistent elevation of glucose-independent insulin secretion suggests that HFD supplementation for 84 days provokes type II diabetes. Furthermore, fat overload in the pancreas dysregulates exocrine function by inducing lipotoxic stress on the endoplasmic reticulum [50], which is in parallel with the present observation that the HFD decreased zymogen granules in the pancreas. In addition to the anti-obesity activity of M-LB/CS, the present results imply that the administration of the three different doses of M-LB/CS not only preserve exocrine function, but also enhance insulin sensitivity by inhibiting the abnormal expansion of pancreatic islets and by decreasing blood levels of glycosylated hemoglobin, insulin, and glucose in mice fed the HFD.

In the present study, we showed that M-LB/CS dose-dependently blocked the HFD-mediated reduction of AMPK mRNA. In response to low-energy states, AMPK has been considered a metabolic guardian by inhibiting anabolic processes and facilitating catabolic processes, mitochondrial biogenesis, and autophagy [26]. In particular, AMPK inhibits fatty acid and cholesterol synthesis through direct inhibition of ACC, SREBP-1, and HMG-CoA reductase [51,52]. In addition, AMPK phosphorylates several transcriptional regulators (e.g., CREB-regulated transcription coactivator 2, class IIa histone deacetylase), which in turn reduce the transcription of gluconeogenic genes (e.g., PEPCK and G6Pase) [53,54]. Therefore, the present results shown for the liver tissue raise the possibility that M-LB/CS alleviated HFD-induced lipid accumulation and gluconeogenesis by activating AMPK. Although we only observed AMPK expression in the hepatic tissue, AMPK has been reported to be ubiquitously expressed in extrahepatic tissues [26,55]. Therefore, M-LB/CS-mediated AMPK activation in conjunction with regulating other signaling molecules (e.g., PPARs) may also contribute to decreased glucose and lipid levels in serum, block lipid accumulation in the fat pad, and reduce lipotoxic vacuoles in the renal tubules.

In the present study, we showed that M-LB/CS not only scavenged DPPH radicals in vitro, but also halted hepatic lipid peroxidation in mice fed the HFD. In addition, M-LB/CS dose-dependently prevented the HFD-mediated reductions in the antioxidant system in the hepatic tissue. Glutamate cysteine ligase (the first and rate-limiting enzyme for glutathione biogenesis), catalase, and superoxide dismutase are representative antioxidant enzymes that are transcriptionally regulated by nuclear factor erythroid 2-related factor 2 (Nrf2) [56]. Interestingly, it has been reported that AMPK, alone or in conjunction with glycogen synthase kinase 3β, transactivates antioxidant genes by phosphorylating the serine 558 residue of human Nrf2 (the serine 550 residue of mouse/rat Nrf2) and accumulating Nrf2 in the nucleus [57]. Although detailed molecular mechanisms need to be further studied, the present results suggest that M-LB/CS reduces HFD-mediated oxidative stress by directly scavenging radicals and possibly by facilitating AMPK-dependent Nrf2 activation.

## 5. Conclusions

**Figure 7:**
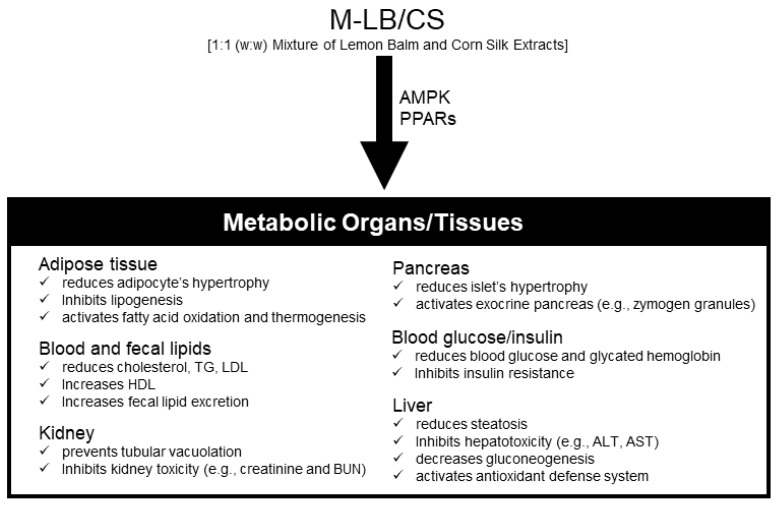
Schematic illustration summarizing the effects of M-LB/CS in HFD-induced metabolic disorders in mice.

The present results demonstrated that M-LB/CS could mitigate HFD-induced obesity in a dosage-dependent manner. In addition, M-LB/CS dose-dependently prevented abnormal lipotoxic changes in the pancreas, liver, and kidney caused by the HFD. The present results suggest that modulation of PPARs and AMPK might contribute to the beneficial effects of M-LB/CS on an HFD (Figure 7). In particular, the effect of 100 mg/kg M-LB/CS on all biomarkers altered by HFD supplementation was comparable to that of 250 mg/kg Met. However, for more than half of the observed biomarkers, the prophylactic effect of 200 mg/kg M-LB/CS-administration was statistically superior to that of Met. Although the present study confirmed the dose-dependent effects of M-LB/CS against HFD-mediated metabolic disorders, several critical steps to the development (e.g., toxicological studies, formulation studies according to consumer preference, and human clinical trials) of M-LB/CS as a nutraceutical remain. If subsequent steps related to M-LB/CS are successfully carried out, M-LB/CS will become a potent nutraceutical to manage obesity-mediated metabolic disorders.

## Figures and Tables

**Table 1:** Oligonucleotide sequences used in the present study.

Gene Name	Sense Primer	Antisense Primer	ReqSeq No.	Amplicon Size (bp)
ACC1	5′-GCCATTGGTATTGGGGCTTAC-3′	5′-CCCGACCAAGGACTTTGTTG-3′	NM_133360.2	112
Adiponectin	5′-CCCAAGGGAACTTGTGCAGGTTGGATG-3′	5′-GTTGGTATCATGGTAGAGAAGAAAGCC-3′	NM_009605.5	639
AMPKα1	5′-AAGCCGACCCAATGACATCA-3′	5′-CTTCCTTCGTACACGCAAAT-3′	NM_001013367.3	103
C/EBPα	5′-TGGACAAGAACAGCAACGAGTAC-3′	5′-CGGTCATTGTCACTGGTCAACT-3′	NM_001287514.1	136
FAS	5′-GCTGCGGAAACTTCAGGAAAT-3′	5′-AGAGACGTGTCACTCCTGGACTT-3′	NM_007988.3	84
GAPDH	5′-CATCTTCCAGGAGCGAGACC-3′	5′-TCCACCACCCTGTTGCTGTA-3′	NM_001289726.1	753
Leptin	5′-CCAAAACCCTCATCAAGACC-3′	5′-GTCCAACTGTTGAAGAATGTCCC-3′	NM_008493.3	394
PPARα	5′-ATGCCAGTACTGCCGTTTTC-3′	5′-GGCCTTGACCTTGTTCATGT-3′	NM_011144.6	220
PPARγ	5′-AGTGGAGACCGCCCAGG-3′	5′-GCAGCAGGTTGTCTTGGATGT-3′	NM_00127330.2	64
SREBP-1c	5′-AGCCTGGCCATCTGTGAGAA-3′	5′-CAGACTGGTACGGGCCACAA-3′	NM_011480.4	132
UCP2	5′-CCGCATTGGCCTCTACGACTCT-3′	5′-CCCCGAAGGCAGAAGTGAAGTG-3′	NM_011671.5	386

ACC, acetyl-CoA carboxylase; AMPK, AMP-activated protein kinase; C/EBP, CC/AAT enhancer-binding protein; FAS, fatty acid synthase; GAPDH, glyceraldehyde 3-phosphate dehydrogenase; PPAR, peroxisome proliferator–activated receptor; SREBP, sterol regulatory element-binding protein; UCP, uncoupling protein.

**Table 2:** Effects of M-LB/CS on blood and fecal biomarkers associated with metabolic disorders.

Sample	Biomarker (Unit)	Experimental Group					
		NFD	HFD	HFD + Met	HFD + M-LB/CS (200 mg/kg)	HFD + M-LB/CS (100 mg/kg)	HFD + M-LB/CS (50 mg/kg)
Serum	Cholesterol (mg/dL)	87.63 ± 15.02	280.50 ± 23.81 ^a^	165.63 ± 18.72 ^a,c^	117.75 ± 22.34 ^c,e^	166.00 ± 20.15 ^a,c^	203.13 ± 19.97 ^a,c,e^
	Triglycerides (mg/dL)	71.00 ± 15.94	238.25 ± 17.53 ^a^	138.50 ± 17.97 ^a,c^	106.50 ± 13.14 ^a,c,f^	134.25 ± 20.43 ^a,c^	184.75 ± 28.45 ^a,c^
	LDL (mg/dL)	16.13 ± 2.80	69.75 ± 12.43 ^a^	39.75 ± 11.49 ^a,c^	28.00 ± 11.55 ^c^	36.75 ± 10.78 ^a,c^	51.00 ± 10.57 ^a,d^
	HDL (mg/dL)	88.88 ± 15.68	22.25 ± 4.46 ^a^	55.88 ± 11.22 ^a,c^	70.38 ± 11.54 ^b,c^	53.38 ± 10.84 ^a,c^	42.63 ± 11.55 ^a,d^
	Insulin (mg/mL)	0.62 ± 0.12	2.51 ± 0.43 ^a^	1.49 ± 0.15 ^a,c^	1.18 ± 0.36 ^b,c^	1.48 ± 0.11 ^a,c^	1.77 ± 0.19 ^a,d^
	ALT (IU/L)	38.13 ± 12.37	161.38 ± 17.70 ^a^	104.38 ± 20.60 ^a,c^	66.63 ± 21.00 ^b,c,f^	105.50 ± 15.80 ^a,c^	125.75 ± 16.56 ^a,c^
	AST (IU/L)	74.50 ± 15.82	221.88 ± 24.52 ^a^	142.63 ± 15.96 ^a,c^	94.63 ± 11.94 ^c,f^	139.00 ± 8.00 ^a,c^	166.50 ± 16.33 ^a,c^
	Creatinine (mg/dL)	0.54 ± 0.17	2.10 ± 0.28 ^a^	1.05 ± 0.27 ^a,c^	0.78 ± 0.17 ^c^	1.03 ± 0.21 ^a,c^	1.43 ± 0.20 ^a,c,f^
	BUN (mg/dL)	28.88 ± 11.12	104.38 ± 15.77 ^a^	52.50 ± 16.30 ^b,c^	41.00 ± 13.77 ^c^	52.75 ± 9.48 ^b,c^	75.88 ± 12.47 ^a,c,f^
Plasma	Glucose (mg/dL)	94.63 ± 23.40	257.13 ± 31.82 ^a^	157.13 ± 22.43 ^a,c^	129.13 ± 24.23 ^b,c^	153.50 ± 17.76 ^a,c^	186.00 ± 14.00 ^a,c^
Blood	Glycosylated hemoglobin (%)	2.94 ± 1.21	9.12 ± 0.80 ^a^	5.36 ± 0.72 ^a,c^	3.77 ± 1.00 ^c,f^	5.32 ± 0.64 ^a,c^	6.71 ± 1.50 ^a,c^
Feces	Cholesterol (mg/g feces)	1.20 ± 0.30	1.36 ± 0.27	3.29 ± 0.48 ^a,c^	4.86 ± 1.25 ^a,c^	3.39 ± 0.49 ^a,c^	2.08 ± 0.63 ^f^
	Triglycerides (mg/g feces)	0.79 ± 0.14	0.82 ± 0.13	3.00 ± 1.09 ^a,c^	3.97 ± 0.62 ^a,c^	2.94 ± 0.62 ^a,c^	1.87 ± 0.64 ^b,d^

^a^ *p* < 0.01, ^b^ *p* < 0.05 vs. NFD; ^c^ *p* < 0.01, ^d^ *p* < 0.05 vs. HFD; ^e^ *p* < 0.01, ^f^ *p* < 0.05 vs. HFD + Met. LDL, low-density lipoprotein; HDL, high-density lipoprotein; ALT, alanine aminotransferase; AST, aspartate aminotransferase; BUN, blood urea nitrogen; IU, international unit.

## Data Availability

Data is contained within the article and Supplementary Materials.

## Supplementary Materials

The following supporting information can be downloaded at: https://www.mdpi.com/article/10.3390/antiox11040730/s1, Figure S1: Effect of M-LB/CS on DPPH radical.

## References

[1] Alberti K.G., Eckel R.H., Grundy S.M., Zimmet P.Z., Cleeman J.I., Donato K.A., Fruchart J.C., James W.P., Loria C.M., Smith S.C. (2009). Harmonizing the metabolic syndrome: A joint interim statement of the International Diabetes Federation Task Force on Epidemiology and Prevention; National Heart, Lung, and Blood Institute; American Heart Association; World Heart Federation; International Atherosclerosis Society; and International Association for the Study of Obesity. Circulation.

[2] Belete R., Ataro Z., Abdu A., Sheleme M. (2021). Global prevalence of metabolic syndrome among patients with type I diabetes mellitus: A systematic review and meta-analysis. Diabetol. Metab. Syndr..

[3] Furukawa S., Fujita T., Shimabukuro M., Iwaki M., Yamada Y., Nakajima Y., Nakayama O., Makishima M., Matsuda M., Shimomura I. (2004). Increased oxidative stress in obesity and its impact on metabolic syndrome. J. Clin. Invest..

[4] Maurizi G., Della Guardia L., Maurizi A., Poloni A. (2018). Adipocytes properties and crosstalk with immune system in obesity-related inflammation. J. Cell. Physiol..

[5] Cani P.D., Amar J., Iglesias M.A., Poggi M., Knauf C., Bastelica D., Neyrinck A.M., Fava F., Tuohy K.M., Chabo C. (2007). Metabolic endotoxemia initiates obesity and insulin resistance. Diabetes.

[6] Tan B.L., Norhaizan M.E. (2019). Effect of High-Fat Diets on Oxidative Stress, Cellular Inflammatory Response and Cognitive Function. Nutrients.

[7] Shakeri A., Sahebkar A., Javadi B. (2016). *Melissa officinalis* L.—A review of its traditional uses, phytochemistry and pharmacology. J. Ethnopharmacol..

[8] Hasanudin K., Hashim P., Mustafa S. (2012). Corn silk (Stigma maydis) in healthcare: A phytochemical and pharmacological review. Molecules.

[9] Kim M., Yoo G., Randy A., Son Y.J., Hong C.R., Kim S.M., Nho C.W. (2020). Lemon Balm and Its Constituent, Rosmarinic Acid, Alleviate Liver Damage in an Animal Model of Nonalcoholic Steatohepatitis. Nutrients.

[10] Asadi A., Shidfar F., Safari M., Hosseini A.F., Fallah Huseini H., Heidari I., Rajab A. (2019). Efficacy of *Melissa officinalis* L. (lemon balm) extract on glycemic control and cardiovascular risk factors in individuals with type 2 diabetes: A randomized, double-blind, clinical trial. Phytother. Res..

[11] Li C.C., Lee Y.C., Lo H.Y., Huang Y.W., Hsiang C.Y., Ho T.Y. (2019). Antihypertensive Effects of Corn Silk Extract and Its Novel Bioactive Constituent in Spontaneously Hypertensive Rats: The Involvement of Angiotensin-Converting Enzyme Inhibition. Molecules.

[12] Wang K.J., Zhao J.L. (2019). Corn silk (*Zea mays* L.), a source of natural antioxidants with α-amylase, α-glucosidase, advanced glycation and diabetic nephropathy inhibitory activities. Biomed. Pharmacother..

[13] Lee E.Y., Kim S.L., Kang H.J., Kim M.H., Ha A.W., Kim W.K. (2016). High maysin corn silk extract reduces body weight and fat deposition in C57BL/6J mice fed high-fat diets. Nutr. Res. Pract..

[14] Cho I.J., Kim S.E., Choi B.R., Park H.R., Park J.E., Hong S.H., Kwon Y.S., Oh W.S., Ku S.K. (2021). Lemon Balm and Corn Silk Extracts Mitigate High-Fat Diet-Induced Obesity in Mice. Antioxidants.

[15] Yerevanian A., Soukas A.A. (2019). Metformin: Mechanisms in Human Obesity and Weight Loss. Curr. Obes. Rep..

[16] Suh J.T., Kim K.D., Sohn H.B., Kim S.J., Hong S.Y., Kim Y.H. (2020). Comparative Study of Antioxidant Activities at Different Cultivation Area and Harvest Date of the Gomchwi ‘Sammany’ Variety. Korean J. Plant. Res..

[17] Choi B.R., Cho I.J., Jung S.J., Kim J.K., Park S.M., Lee D.G., Ku S.K., Park K.M. (2020). Lemon balm and dandelion leaf extract synergistically alleviate ethanol-induced hepatotoxicity by enhancing antioxidant and anti-inflammatory activity. J. Food Biochem..

[18] Kim C.M., Yi S.J., Cho I.J., Ku S.K. (2013). Red-koji fermented red ginseng ameliorates high fat diet-induced metabolic disorders in mice. Nutrients.

[19] Schmittgen T.D., Livak K.J. (2008). Analyzing real-time PCR data by the comparative C(T) method. Nat. Protoc..

[20] Stio M., Vanni P., Pinzauti G. (1988). A continuous spectrophotometric assay for the enzymatic marker glucose 6-phosphatase. Anal. Biochem..

[21] Cha J.H., Kim S.R., Kang H.J., Kim M.H., Ha A.W., Kim W.K. (2016). Corn silk extract improves cholesterol metabolism in C57BL/6J mouse fed high-fat diets. Nutr. Res. Pract..

[22] Koska J., Stefan N., Permana P.A., Weyer C., Sonoda M., Bogardus C., Smith S.R., Joanisse D.R., Funahashi T., Krakoff J. (2008). Increased fat accumulation in liver may link insulin resistance with subcutaneous abdominal adipocyte enlargement, visceral adiposity, and hypoadiponectinemia in obese individuals. Am. J. Clin. Nutr..

[23] Heydemann A. (2016). An Overview of Murine High Fat Diet as a Model for Type 2 Diabetes Mellitus. J. Diabetes Res..

[24] Grossman A. (1984). An overview of pancreatic exocrine secretion. Comp. Biochem. Physiol. B..

[25] Shirwany N.A., Zou M.H. (2014). AMPK: A cellular metabolic and redox sensor. A minireview. Front. Biosci..

[26] Herzig S., Shaw R.J. (2018). AMPK: Guardian of metabolism and mitochondrial homeostasis. Nat. Rev. Mol. Cell Biol..

[27] Zhang X., Yang S., Chen J., Su Z. (2019). Unraveling the Regulation of Hepatic Gluconeogenesis. Front. Endocrinol..

[28] Maksimović Z., Malenović A., Jancić B., Kovacević N. (2004). Quantification of allantoin in various *Zea mays* L. hybrids by RP-HPLC with UV detection. Pharmazie.

[29] Luo C., Sun H., Peng J., Gao C., Bao L., Ji R., Zhang C., Zhu W., Jin Y. (2021). Rosmarinic acid exerts an antagonistic effect on nonalcoholic fatty liver disease by regulating the YAP1/TAZ-PPARγ/PGC-1α signaling pathway. Phytother. Res..

[30] Nyandwi J.B., Ko Y.S., Jin H., Yun S.P., Park S.W., Kim H.J. (2021). Rosmarinic Acid Exhibits a Lipid-Lowering Effect by Modulating the Expression of Reverse Cholesterol Transporters and Lipid Metabolism in High-Fat Diet-Fed Mice. Biomolecules.

[31] Ma J., Meng X., Liu Y., Yin C., Zhang T., Wang P., Park Y.K., Jung H.W. (2020). Effects of a rhizome aqueous extract of Dioscorea batatas and its bioactive compound, allantoin in high fat diet and streptozotocin-induced diabetic mice and the regulation of liver, pancreas and skeletal muscle dysfunction. J. Ethnopharmacol..

[32] Govindaraj J., Sorimuthu Pillai S. (2015). Rosmarinic acid modulates the antioxidant status and protects pancreatic tissues from glucolipotoxicity mediated oxidative stress in high-fat diet: Streptozotocin-induced diabetic rats. Mol. Cell. Biochem..

[33] Domitrović R., Potočnjak I., Crnčević-Orlić Z., Škoda M. (2014). Nephroprotective activities of rosmarinic acid against cisplatin-induced kidney injury in mice. Food Chem. Toxicol..

[34] Chung H.H., Lee K.S., Cheng J.T. (2013). Decrease of Obesity by Allantoin via Imidazoline I 1 -Receptor Activation in High Fat Diet-Fed Mice. Evid. Based Complement. Alternat. Med..

[35] Kumar S., Pandey A.K. (2013). Chemistry and biological activities of flavonoids: An overview. Sci. World J..

[36] Chen H., Nie T., Zhang P., Ma J., Shan A. (2022). Hesperidin attenuates hepatic lipid accumulation in mice fed high-fat diet and oleic acid induced HepG2 via AMPK activation. Life Sci..

[37] Li J., Wang T., Liu P., Yang F., Wang X., Zheng W., Sun W. (2021). Hesperetin ameliorates hepatic oxidative stress and inflammation via the PI3K/AKT-Nrf2-ARE pathway in oleic acid-induced HepG2 cells and a rat model of high-fat diet-induced NAFLD. Food Funct..

[38] Wu L., Guo T., Deng R., Liu L., Yu Y. (2021). Apigenin Ameliorates Insulin Resistance and Lipid Accumulation by Endoplasmic Reticulum Stress and SREBP-1c/SREBP-2 Pathway in Palmitate-Induced HepG2 Cells and High-Fat Diet-Fed Mice. J. Pharmacol. Exp. Ther..

[39] Yang Y., Wu Y., Zou J., Wang Y.H., Xu M.X., Huang W., Yu D.J., Zhang L., Zhang Y.Y., Sun X.D. (2021). Naringenin Attenuates Non-Alcoholic Fatty Liver Disease by Enhancing Energy Expenditure and Regulating Autophagy via AMPK. Front. Pharmacol..

[40] Jiang H., Horiuchi Y., Hironao K.Y., Kitakaze T., Yamashita Y., Ashida H. (2020). Prevention effect of quercetin and its glycosides on obesity and hyperglycemia through activating AMPKα in high-fat diet-fed ICR mice. J. Clin. Biochem. Nutr..

[41] Lee C.W., Seo J.Y., Kim S.L., Lee J., Choi J.W., Park Y.I. (2017). Corn silk maysin ameliorates obesity in vitro and in vivo via suppression of lipogenesis, differentiation, and function of adipocytes. Biomed. Pharmacother..

[42] Xu N., Zhang L., Dong J., Zhang X., Chen Y.G., Bao B., Liu J. (2014). Low-dose diet supplement of a natural flavonoid, luteolin, ameliorates diet-induced obesity and insulin resistance in mice. Mol. Nutr. Food Res..

[43] Pu P., Gao D.M., Mohamed S., Chen J., Zhang J., Zhou X.Y., Zhou N.J., Xie J., Jiang H. (2012). Naringin ameliorates metabolic syndrome by activating AMP-activated protein kinase in mice fed a high-fat diet. Arch. Biochem. Biophys..

[44] Rosen E.D., Hsu C.H., Wang X., Sakai S., Freeman M.W., Gonzalez F.J., Spiegelman B.M. (2002). C/EBPalpha induces adipogenesis through PPARgamma: A unified pathway. Genes Dev..

[45] Hong F., Pan S., Guo Y., Xu P., Zhai Y. (2019). PPARs as Nuclear Receptors for Nutrient and Energy Metabolism. Molecules.

[46] Ahmadian M., Suh J.M., Hah N., Liddle C., Atkins A.R., Downes M., Evans R.M. (2013). PPARγ signaling and metabolism: The good, the bad and the future. Nat. Med..

[47] Weidner C., Wowro S.J., Freiwald A., Kodelja V., Abdel-Aziz H., Kelber O., Sauer S. (2014). Lemon balm extract causes potent antihyperglycemic and antihyperlipidemic effects in insulin-resistant obese mice. Mol. Nutr. Food Res..

[48] Wali J.A., Jarzebska N., Raubenheimer D., Simpson S.J., Rodionov R.N., O’Sullivan J.F. (2020). Cardio-Metabolic Effects of High-Fat Diets and Their Underlying Mechanisms-A Narrative Review. Nutrients.

[49] Larsen M.L., Hørder M., Mogensen E.F. (1990). Effect of long-term monitoring of glycosylated hemoglobin levels in insulin-dependent diabetes mellitus. N. Engl. J. Med..

[50] Danino H., Ben-Dror K., Birk R. (2015). Exocrine pancreas ER stress is differentially induced by different fatty acids. Exp. Cell Res..

[51] Li Y., Xu S., Mihaylova M.M., Zheng B., Hou X., Jiang B., Park O., Luo Z., Lefai E., Shyy J.Y. (2011). AMPK phosphorylates and inhibits SREBP activity to attenuate hepatic steatosis and atherosclerosis in diet-induced insulin-resistant mice. Cell Metab..

[52] Carling D., Zammit V.A., Hardie D.G. (1987). A common bicyclic protein kinase cascade inactivates the regulatory enzymes of fatty acid and cholesterol biosynthesis. FEBS Lett..

[53] Mihaylova M.M., Vasquez D.S., Ravnskjaer K., Denechaud P.D., Yu R.T., Alvarez J.G., Downes M., Evans R.M., Montminy M., Shaw R.J. (2011). Class IIa histone deacetylases are hormone-activated regulators of FOXO and mammalian glucose homeostasis. Cell.

[54] Lee J.M., Seo W.Y., Song K.H., Chanda D., Kim Y.D., Kim D.K., Lee M.W., Ryu D., Kim Y.H., Noh J.R. (2010). AMPK-dependent repression of hepatic gluconeogenesis via disruption of CREB.CRTC2 complex by orphan nuclear receptor small heterodimer partner. J. Biol. Chem..

[55] Verhoeven A.J., Woods A., Brennan C.H., Hawley S.A., Hardie D.G., Scott J., Beri R.K., Carling D. (1995). The AMP-activated protein kinase gene is highly expressed in rat skeletal muscle. Alternative splicing and tissue distribution of the mRNA. Eur. J. Biochem..

[56] Kobayashi M., Yamamoto M. (2006). Nrf2-Keap1 regulation of cellular defense mechanisms against electrophiles and reactive oxygen species. Adv. Enzyme Regul..

[57] Joo M.S., Kim W.D., Lee K.Y., Kim J.H., Koo J.H., Kim S.G. (2016). AMPK Facilitates Nuclear Accumulation of Nrf2 by Phosphorylating at Serine 550. Mol. Cell. Biol..

