# Supplementary material for: Lemon Balm and Corn Silk Mixture Alleviates Metabolic Disorders Caused by a High-Fat Diet
Source: Antioxidants (Basel). 2022 Apr 7;11(4):730. doi: 10.3390/antiox11040730 (PMC9029851; doi:10.3390/antiox11040730)
Supplement: Supplementary file 1 [file antioxidants-11-00730-s001.zip › antioxidants-1666467-supplementary.pdf]

antioxidants

MDPI

Supplementary information

Lemon Balm and Corn Silk Mixture Alleviates Metabolic Disorders Caused by a High-Fat Diet

Il-Je Cho [1,†], Joung-Hoon Shin [2,†], Beom-Rak Choi [3], Hye-Rim Park [3], Jeong-Eun Park [3], Seong-Hwa Hong [3], Young-Sam Kwon [2], Won-Seok Oh [4,*] and Sae-Kwang Ku [1,*]

1 Department of Preparatory Korean Medicine, College of Korean Medicine, Daegu Haany University, Gyeongsan, Gyeongsangbuk-do 38610, Korea; skek023@gmail.com
2 Department of Veterinary Surgery, College of Veterinary Medicine, Kyungpook National University, Daegu 41566, Korea; newyorkah@knu.ac.kr (J.-H.S.), kwon@knu.ac.kr (Y.-S.K.)
3 Nutracore Co., Ltd., Gwanggyo SK Viewlake A-3206, Beobjo-Ro 25, Suwon, Gyeonggi-do 16514, Korea; brchoi@nutracore.co.kr (B.-R.C.), hrpark@nutracore.co.kr (H.-R.P.), jpark@nutracore.co.kr (J.-E.P.), shhong@nutracore.co.kr (S.-H.H.)
4 Department of Veterinary Internal Medicine, College of Veterinary Medicine, Kyungpook National University, Daegu 41566, Korea
* Correspondence: owsvcs@hanmail.net (W.-S.O.); gucci200@dhu.ac.kr (S.-K.K.); Tel.: +82-53-950-5951 (W.-S.O.); +82-53-819-1549 (S.-K.K.)
† Contributed equally to this work

Supplementary Materials and Methods

Measurement of Radical Scavenging Activity

Radical scavenging activity of M-LB/CS was determined using a 2,2-Diphenyl-1-picrylhydrazyl (DPPH), as described previously [1]. After incubating 180 µL of DPPH (150 µM) with various concentration of M-LB/CS (20 µL each) for 30 min in the dark, absorbance at 517 nm was determined using an EnSpire™ multimode plate reader (PerkinElmer, Waltham, MA, USA). DPPH radical intensity was calculated as a percentage of the vehicle.

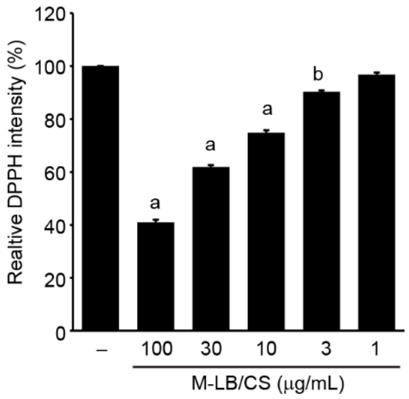

Figure S1. Effect of M-LB/CS on DPPH radical: [a] $P < 0.01$, [b] $P < 0.05$ versus vehicle.

Supplementary References

1. Cho, I.J.; Kim, S.E.; Choi, B.R.; Park, H.R.; Park, J.E.; Hong, S.H.; Kwon, Y.S.; Oh, W.S.; Ku, S.K. Lemon Balm and Corn Silk Extracts Mitigate High-Fat Diet-Induced Obesity in Mice. *Antioxidants (Basel)* **2021**, *10*, 2015. doi: 10.3390/antiox10122015.
